# The Three Essential Motifs in P0 for Suppression of RNA Silencing Activity of *Potato leafroll virus* Are Required for Virus Systemic Infection

**DOI:** 10.3390/v11020170

**Published:** 2019-02-20

**Authors:** Mamun-Or Rashid, Xiao-Yan Zhang, Ying Wang, Da-Wei Li, Jia-Lin Yu, Cheng-Gui Han

**Affiliations:** State Key Laboratory for Agrobiotechnology and Key Laboratory of Pest Monitoring and Green Management, MOA, China Agricultural University, Beijing 100193, China; mamun_1961@yahoo.com (M.-O.R.); xiaoyan433@cau.edu.cn (X.-Y.Z.); yingwang@cau.edu.cn (Y.W.); lidw@cau.edu.cn (D.-W.L.); yjl@cau.edu.cn (J.-L.Y.)

**Keywords:** *Potato leafroll virus*, viral suppressor of RNA silencing, P0 mutation, F-box-like motif, G/W/G-like motif, C-terminal conserved region, virus infection

## Abstract

Higher plants exploit posttranscriptional gene silencing as a defense mechanism against virus infection by the RNA degradation system. Plant RNA viruses suppress posttranscriptional gene silencing using their encoded proteins. Three important motifs (F-box-like motif, G139/W140/G141-like motif, and C-terminal conserved region) in P0 of *Potato leafroll virus* (PLRV) were reported to be essential for suppression of RNA silencing activity. In this study, *Agrobacterium-*mediated transient experiments were carried out to screen the available amino acid substitutions in the F-box-like motif and G139/W140/G141-like motif that abolished the RNA silencing suppression activity of P0, without disturbing the P1 amino acid sequence. Subsequently, four P0 defective mutants derived from a full-length cDNA clone of PLRV (L76F and W87R substitutions in the F-box-like motif, G139RRR substitution in the G139/W140/G141-like motif, and F220R substitution in the C-terminal conserved region) were successfully generated by reverse PCR and used to investigate the impact of these substitutions on PLRV infectivity. The RT-PCR and western blot analysis revealed that these defective mutants affected virus accumulation in inoculated leaves and systemic movement in *Nicotiana benthamiana* as well as in its natural hosts, potato and black nightshade. These results further demonstrate that the RNA silencing suppressor of PLRV is required for PLRV accumulation and systemic infection.

## 1. Introduction

Potato (*Solanum tuberosum*) is grown worldwide, and is considered the fourth most important staple food source after rice (*Oryza sativa*), maize (*Zea mays*), and wheat (*Triticum aestivum*) [1,2]. Globally, ~376,826,967 tonnes of potatoes were produced from ~19,246,462 ha of land in 2016 [3], and they are consumed by over one billion people. More than 40 viruses and two viroids can infect potatoes [4]. Among them, *Potato leafroll virus* (PLRV) is one of the most economically important and destructive viral diseases. It causes individual plant yield losses of over 50%, which leads to an annual global yield loss of 20 million tonnes [5]. Highly susceptible varieties may have yield losses of up to 90% and quality can be diminished when cultivars develop symptoms like net necrosis [6]. PLRV is the representative member of the genus *Polerovirus* in the family Luteoviridae [7] and is transmitted by aphid vectors, mostly by the green peach aphid *Myzus persicae* (Homoptera: Aphididae) in a circulative, non-propagative way and is confined to the phloem tissues of the infected plant [8,9]. It is a positive sense, single-stranded RNA virus that contains a genome of approximately 5.9 kb with six open reading frames (ORFs) encoding six proteins [10,11]. Its ORF0 encodes a 28-kDa silencing suppressor called P0 protein, which is responsible for symptom development [12]. RNA silencing is a natural defense mechanism of hosts against viral infections at the nucleic acid level [13,14], which is initiated when double-stranded RNAs are processed by dicer-like enzymes to produce complementary short (21–24 nucleotides (nt)) RNAs, called small interfering RNAs (siRNAs) [15]. Numerous viral-encoded suppressors of RNA silencing (VSR) proteins have evolved to overcome host RNA silencing [14,16,17,18,19]. The best-characterized VSR employing this strategy is *Tomato bushy stunt virus* (TBSV) P19 protein, that binds siRNA duplexes [20]. Sequestration of siRNA is the most common mode of action of RNA silencing suppressors [21,22,23]. Another mechanism of RNA silencing suppressors is through protein–protein interaction [24]. The P0 proteins encoded by the 5′-proximal ORF of the *Beet mild yellowing virus*, *Cotton leafroll dwarf virus*, *Cucurbit aphid-borne yellows virus* (CABYV), *Melon aphid-borne yellows virus*, *Sugarcane yellow leaf virus*, *Turnip yellows virus* (TuYV, synonyms *Beet western yellows virus* FL strain, BWYV-FL), *Wheat yellow dwarf virus-*GPV isolate and PLRV of the genus *Polerovirus* have been reported to suppress RNA silencing [25,26,27,28,29,30,31]. The P0 protein can generate cell death within the infiltration region in *Nicotiana* species [12,24,32]. The F-box-like motifs of P0 protein of TuYV (P0^Tu^, formerly P0^BW^) and CABYV (P0^CA^) have been proposed to suppress RNA silencing by interacting with S-phase kinase-related protein-1 (SKP1), a subunit of the SCF family of E3 ubiquitin ligases [33], where they take part in the ubiquitination and degradation of Argonaute1 (AGO1) [12,24]. However, this AGO1 degradation by P0 is obstructed by the reticence of autophagy [34], but not by an inhibitor of proteasomes [35]. In addition to the F-box-like motif, a G139/W140/G141-like motif and a C-terminal conserved P0 sequence have vital roles in suppression of RNA silencing [12,26,30]. Exploration of the biological activity of different viral proteins became easier with the availability of infectious full-length cDNA and an agroinfiltration vector to inoculate plants for infection experiments [36,37]. Mutations in the P0 sequence of TuYV (formerly BWYV-FL) actively reduce or eliminate viral RNA accumulation in plants [38].

Zhuo et al. observed that suppressor activity of P0 protein of PLRV (P0^PL^) is eliminated by L76A, W87A, or G88A substitution in the F-box-like motif between 76 and 95 residues and is weakened by W140A substitution in the G139/W140/G141-like motif, as well as by F220R substitution in the C-terminal conserved region [30]. However, the effect of these VSR defective mutants on PLRV infection was not addressed. Therefore, to analyze the infectivity of PLRV in *Nicotiana benthamiana* as well as in its natural hosts, we constructed L76F, W87R, G139RRR, and F220R substitution mutants in the above-mentioned three essential conserved regions of full-length cDNA of PLRV that eliminate the suppressor activity of P0 and have no impact on the P1 protein coding. The inoculation assay demonstrated that all VSR defective mutants affected virus accumulation and systemic infection, further confirming that VSR functional P0 is required for PLRV local and systemic infection.

## 2. Materials and Methods

### 2.1. Plant Material and Growth Conditions

Wild-type and GFP transgenic *N. benthamiana* line 16c plants were grown at 24 ± 1 °C with a photoperiod of 16-/8-h light/dark cycle. Potato (cultivar Lalpakri) and black nightshade (*Solanum nigrum*) plants were grown in a greenhouse maintaining the above-mentioned growth conditions.

### 2.2. Plasmid Construction

The binary expression vectors pGD, pGDG, pGDP19 [39], and P0^PL^ [30] were used for transient experiments. The P0^PL^ mutants were constructed from P0^PL^ by reverse-PCR amplification using a specific forward primer containing a *Xho* I restriction site and reverse primers containing an *Apa* I restriction site (Table 1). The resulting DNA fragments, as well as wild-type P0^PL^, were digested with *Xho* I and *Apa* I and inserted into the flag-tagged pGD vector predigested with *Xho* I and *Apa* I.

Full-length infectious cDNA of PLRV was modified with a binary vector pCB301 containing 2× 35S promoter, ribozyme, and NOS terminator from a Canadian isolate of PLRV, pBNUP110 [40]. Briefly, pBNUP110 was amplified with the primer pair PLRV5-28F and PLRVKp3R and the purified DNA fragments were digested with *Kpn* I, which were ligated with pCass4-Rz predigested with *Stu* I and *Kpn* I to produce pCB-PLRV. This pCB-PLRV was amplified with the primer pair PLRV5-28F and PLRVBg3R to obtain pCB-PLRV with *Bgl* II restriction site. The purified DNA was digested with *Bgl* II which was ligated with pCB301 vector predigested with *Stu* I and *Bam*H I to produce the PLRV full-length cDNA clone, referred to as pCB-PLRV. To obtain substitution mutants, pCB-PLRV was amplified using specific forward primers containing *Not* I or *Apa* I restriction sites and reverse primers containing *Apa* I or *Spe* I restriction sites. Resulting PCR products were ligated with pMD19-T (simple) vector (TaKaRa, Shiga, Japan) to produce pTPLRV. Full-length pCB-PLRV mutants were constructed by reverse PCR of this pTPLRV with specific primers (Table 1). The resulting DNA fragments were digested with *Not* I and *Apa* I, or *Apa* I and *Spe* I, and then inserted into the pCB vector predigested with *Not* I and *Apa* I, or *Apa* I and *Spe* I to generate target mutants. All constructs were confirmed by PCR and subsequent sequencing.

### 2.3. Plant Agroinfiltration

Empty pCB301 vector, pCB-PLRV (wild-type), pCB-PLRV derived mutants, _WT_P0^PL^, P0^PL^ derived mutants, empty pGD vector, pGDG, and pGDP19 were transformed into *Agrobacterium tumefaciens* strain C58CI using the freeze–thaw method [41]. The recombinant C58CI was grown overnight at 28 °C, resuspended in infiltration buffer (10 mM MgCl_2_, 10 mM MES, and 100 μM acetosyringone), and raised at room temperature for at least 3 h before infiltration. *Agrobacterium* suspensions were infiltrated into *N. benthamiana* leaves at the six-leaf stage (7–8 weeks old) for transient expression and four-leaf stage (4–5 weeks old) for infectivity analysis, as described earlier [42]. Infectivity was also analyzed by agroinfiltration of 4- to 5-week-old potato and black nightshade plants, the natural hosts for PLRV. Concentration of the cell suspension was measured by spectrophotometry and the optical density was adjusted to 0.5 for each culture. Infiltrated leaves were collected at 3 days post infiltration (dpi) and systemic leaves were collected at 14 dpi for viral RNA and protein analysis. A 100-W, hand-held, long-wave ultraviolet lamp (Black Ray model B 100AP/R; UV Products, Upland, CA, USA) was used to illuminate the plants for photography and a Canon (EOS 550D, Tokyo, Japan) digital camera was used to take photographs.

### 2.4. RNA Extraction and RT-PCR Detection

Total RNAs from the infiltrated plants were extracted by sodium dodecyl sulfate (SDS)–phenol–chloroform method [43]. Briefly, 0.1-g leaf samples were crushed to fine powder in liquid nitrogen. Then, 600 µL of phenol–chloroform mixture and 630 µL of extraction buffer (20 mM Tris–HCl, pH 7.8, 1% sodium dodecyl sulfate, 200 mM sodium chloride, and 5 mM EDTA) were added to them before defrosting with continuous homogenization. The RNA mixed supernatant was separated by centrifugation and the RNAs were precipitated by an equal volume of 4 M lithium chloride and washed with chilled 75% ethanol, followed by chilled 100% ethanol. Pelleted RNAs were dissolved in 40 µL of diethyl pyrocarbonate-treated water. The cDNAs were synthesized by taking 2 µL of these RNAs using specific primer and Moloney murine leukemia virus (M-MLV) reverse transcriptase (Promega, Fitchburg, WI, USA), according to the manufacturer’s instructions. The PCR amplification was performed as previously described [44] using synthesized cDNA as a template with specific primers (Table 1). Amplified products were electrophoresed in 1.0% agarose gel containing ethidium bromide and visualized by UV illumination using a gel documentation system (Gel Doc XR + Imaging System; Bio-Rad, Hercules, CA, USA).

### 2.5. Protein Extraction and Western Blot Analysis

Protein extraction and western blot analysis were performed as described previously [30]. Briefly, total proteins were extracted from 0.1-g leaf samples by grinding into fine powder in liquid nitrogen, followed by adding 300 µL of 2 × SDS buffer (100 mM Tris (pH 6.8), 4% (w/v) SDS, 20% (v/v) glycerol, and 0.2% (w/v) bromophenol blue) with subsequent heating at 100 °C for 10 min. Proteins were separated in 12.5% SDS polyacrylamide gel by electrophoresis and transferred to nitrocellulose membrane (GE Healthcare, Buckinghamshire, UK) using a mini trans-blot electrophoretic transfer cell (Bio-Rad).

Western blot analysis was performed using the rabbit anti-flag antibody (Sigma-Aldrich, St Louis, MO, USA) diluted at 1:1000, or mouse anti-flag antibody (Sigma-Aldrich) diluted at 1:5000, or rabbit anti-GFP antibody (GenScript, Nanjing, China) diluted at 1:3000, or rabbit anti-PLRV movement protein (MP) antibody [45] diluted at 1:5000, as a primary antibody. Incubation was then performed with goat anti-rabbit IgG (Sigma-Aldrich) diluted at 1:3000, or goat anti-mouse HRP (Bio-Rad) diluted at 1:3000, as a secondary antibody. Finally, the membranes were detected using the nitro-blue tetrazolium and 5-bromo-4-chloro-3-indolyphosphate (Sigma-Aldrich), or with a superior chemiluminescence detection kit (GE Healthcare) according to the manufacturer’s instructions.

## 3. Results

### 3.1. Available Amino Acid Substitutions in P0 Essential Motifs that Abolished VSR Activity, without Disturbing P1 Amino Acid Sequence

**Figure 1:**
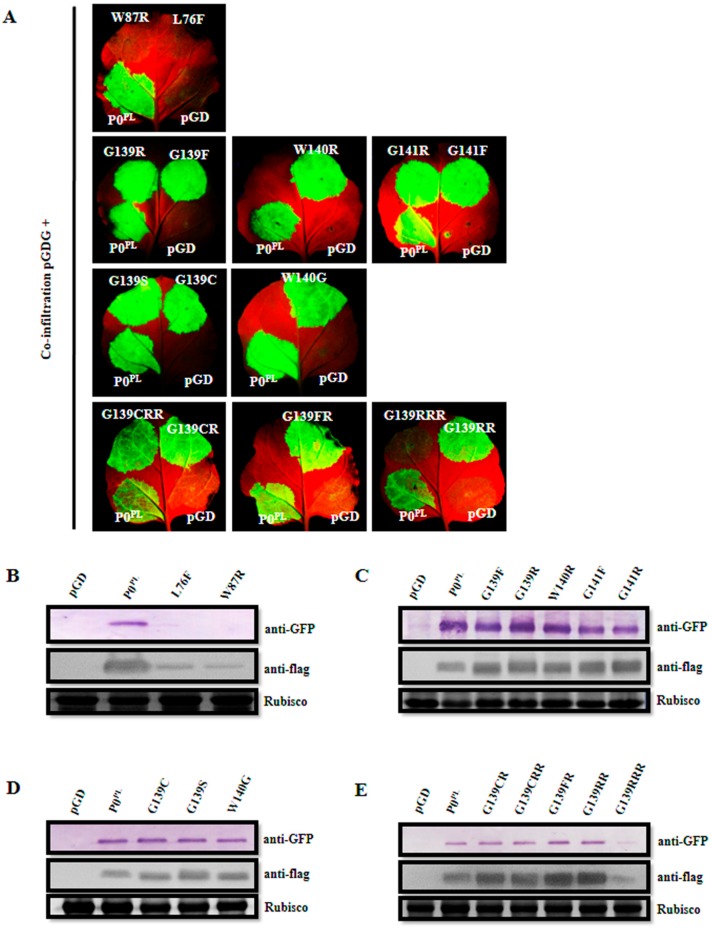
Suppression of local RNA silencing by the mutants of P0 protein of PLRV (P0^PL^) in F-box-like motif and G139/W140/G141-like motif. (**A**) Evaluation of suppression of local RNA silencing in wild-type *Nicotiana benthamiana* leaves co-infiltrated with *Agrobacterium* harboring pGDG and empty pGD vector, or P0^PL^ (wild-type), or P0 mutants, at 3 dpi under long-wavelength UV light. The bottom-right side of leaves shows GFP expression for pGDG plus pGD, the bottom-left side of leaves shows GFP expression for pGDG plus P0^PL^, and both top-right and left side of the leaves show GFP expression for pGDG plus P0^PL^ mutants. (**B**–**E**) Western blot analyses of proteins extracted from the infiltrated leaf patches at 3 dpi using the specific antibody for GFP (anti-GFP) and flag (anti-flag). Rubisco stained with Coomassie brilliant blue was used as a loading control and is shown in the lower panel.

**Figure 2:**
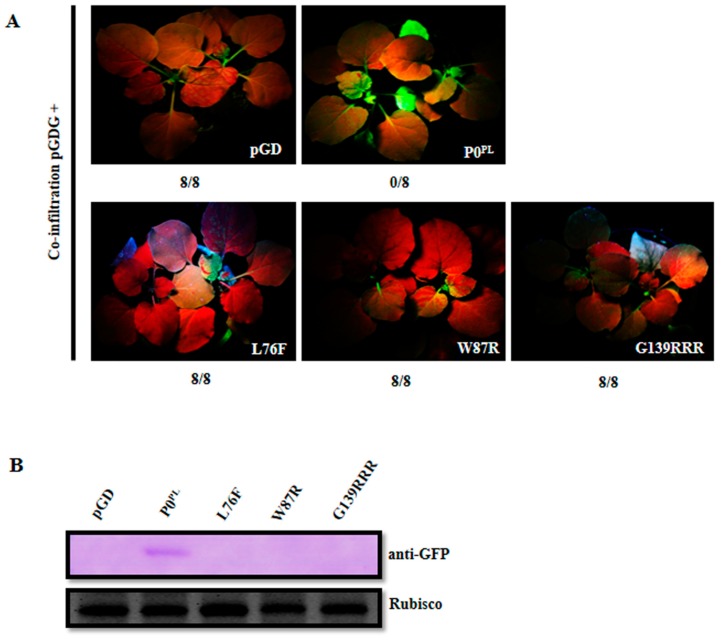
Suppression of systemic RNA silencing by the P0^PL^ mutants in F-box-like motif and G139/W140/G141-like motif. (**A**) Evaluation of suppression of systemic RNA silencing in the *N. benthamiana* 16c line co-infiltrated with *Agrobacterium* harboring pGDG plus empty pGD vector (negative control), or P0^PL^ (wild-type), or P0 mutants, at 14 dpi under long-wavelength UV light. Designations are given at the bottom-right corner of the photographs showing GFP expression in systemic leaves and the bottom numbers of each treatment indicate silencing ratios. (**B**) Western blot analyses of the proteins extracted from systemic leaves of *N. benthamiana* 16c plants co-infiltrated with pGDG plus empty pGD vector, wild-type P0^PL^, L76F, W87R, and G139RRR substitutions respectively, at 14 dpi using GFP specific antibody (anti-GFP). Rubisco stained with Coomassie brilliant blue was used as a loading control and is shown in the lower panel.

Zhuo et al. obtained substitutions in the P0^PL^ essential motifs required for the VSR activity [30] but disturbed the P1 amino acid sequence, except for F220R substitution in the C-terminal conserved region. Here, we need to screen available amino acid substitutions in the P0 essential motifs that have no impact on the P1 amino acid sequence for further modification of full-length infectious cDNA of PLRV. We constructed two substitutions L76F and W87R in the F-box-like motif, eight single amino acid substitutions (G139F, G139R, W140R, G141F, G141R, G139C, G139S, and W140G), three double amino acid substitutions (G139CR, G139FR, and G139RR) and two triple amino acid substitutions (G139CRR and G139RRR) in the G139/W140/G141-like motif (Table S1). The *N. benthamiana* wild-type and *N. benthamiana* 16c leaves were co-infiltrated with *Agrobacterium* harboring pGDG with empty pGD vector, or P0^PL^, or P0 mutants, to evaluate the suppressor activity, whereas empty pGD vector was a negative control and P0^PL^ was a positive control. The *N. benthamiana* leaf patches co-infiltrated with pGDG and all the substitutions in the G139/W140/G141-like motif, except G139RRR, expressed GFP fluorescence as strong as wild-type P0^PL^ on the respective leaf patches at 3 dpi, whereas GFP fluorescence was negligible in the case of co-infiltration with pGDG and L76F, or W87R, or G139RRR substitutions, or the pGD negative control (Table S1, Figure 1A). Western blot analysis from the respective leaf patches at 3 dpi further confirmed that L76F, W87R, and G139RRR substitutions did not accumulate any GFP and a negligible amount of proteins accumulated, whereas all other substitutions in the G139/W140/G141-like motif accumulated GFP and proteins similar to that of P0^PL^ (Figure 1B–E). These results indicated that L76F and W87R substitutions in the F-box-like motif and G139RRR substitution in the G139/W140/G141-like motif abolished the local RNA silencing suppressor activity. Regarding systemic RNA silencing suppression activity, *Agrobacterium* co-infiltrated *N. benthamiana* 16c leaves with pGDG and P0^PL^ expressed a strong GFP fluorescence in upper leaves at 14 dpi, whereas GFP fluorescence was negligible in the case of co-infiltration with pGDG and L76F, or W87R, or G139RRR substitutions, or the pGD negative control (Table S1, Figure 2A). Western blot analysis from the systemic leaves of the respective plants at 14 dpi revealed no accumulation of GFP (Figure 2B), indicating that L76F and W87R substitutions in the F-box-like motif and G139RRR substitution in the G139/W140/G141-like motif also abolished systemic RNA silencing suppressor activity. Similarly, we confirmed that F220R substitution in the C-terminal conserved region abolished systemic RNA silencing suppressor activity (Table S1).

**Figure 3:**
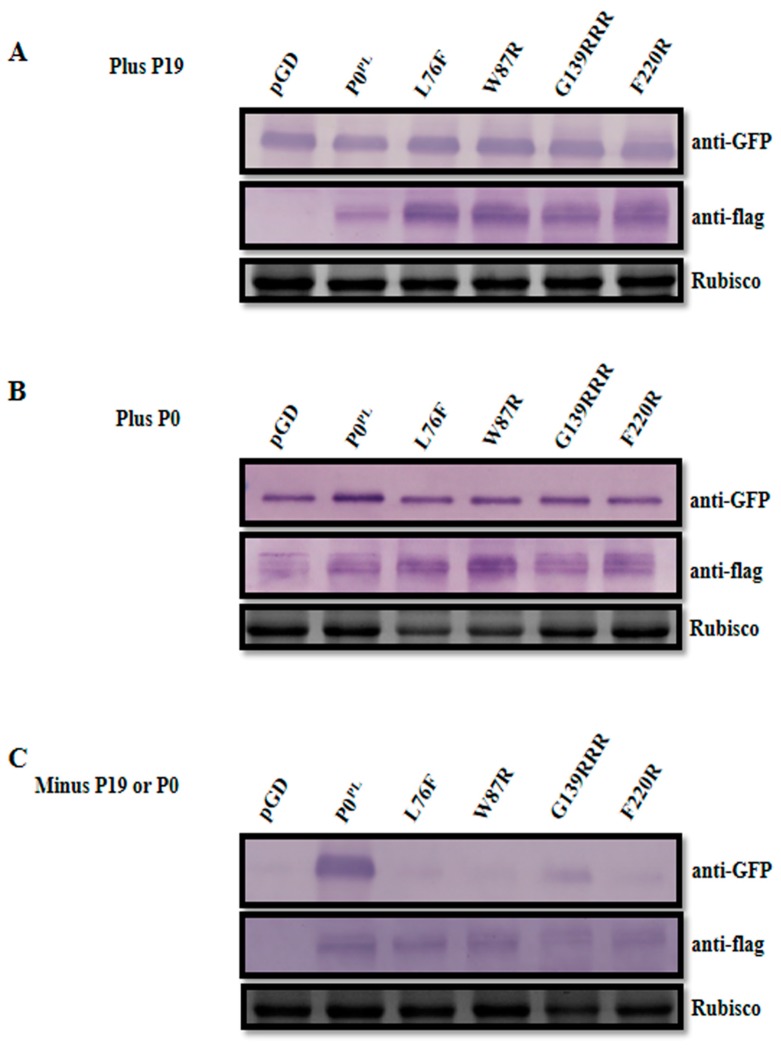
Western blot analysis showing accumulation of GFP and flag-fused P0^PL^ mutants in *N. benthamiana* leaves. Proteins were extracted at 3 dpi from the *N. benthamiana* leaves co-infiltrated with pGDG plus empty pGD vector (negative control), P0^PL^ (wild-type), and L76F, W87R, and G139RRR mutants respectively, in the presence or absence of the *Tomato bushy stunt virus* (TBSV) P19 protein or P0 protein of *Potato leafroll virus*. Rubisco stained with Coomassie brilliant blue was used as a loading control as is shown in the lower panel. (**A**) Plus P19; (**B**) plus P0^PL^; (**C**) minus P19 or P0^PL^.

To compare the protein accumulation levels of these P0^PL^ mutants, *N. benthamiana* leaves were further co-infiltrated with P19 or P0^PL^ and pGDG, as well as the mutant derivatives (L76F and W87R in the F-box-like motif and G139RRR in the G139/W140/G141-like motif). At 3 dpi of co-infiltration with P19 or P0^PL^ and pGDG, respective leaf patches for all mutant derivatives and wild-type P0^PL^ expressed strong GFP fluorescence as well as the empty pGD vector and all the P0 mutants accumulated a high level of protein along with the wild-type P0^PL^ (Figure 3A,B). However, in the absence of P19 or P0^PL^, only leaf patches co-infiltrated with pGDG and wild-type P0^PL^ exhibited GFP fluorescence and protein accumulation at 3 dpi, whereas leaf patches co-infiltrated with pGDG and the P0 mutants were unable to express GFP and only trace amounts of the dysfunctional mutant proteins accumulated in the P0 mutants (Figure 3C). These results further confirmed that the mutant proteins were expressed at a similar extent to wild-type P0^PL^ when RNA silencing was suppressed by P19 or P0^PL^.

### 3.2. Modification of Full-Length Infectious cDNA Clone of PLRV and Generation of its VSR Defective Mutants

The pCB-PLRV containing full-length cDNA of PLRV was constructed by subcloning of pBNUP110 into a binary vector pCass4-Rz. Then, PLRV cDNA was further subcloned into another smaller binary expression vector pCB301 to produce pCB-PLRV. Infectivity of pCB-PLRV and pCB-PLRV were confirmed by RT-PCR using systemic leaves at 14 dpi from agroinfiltrated *N. benthamiana* plants (Figure S1). Based on the above-mentioned screening of available substitutions in the P0, four full-length cDNA mutants of pCB-PLRV (pCB-PL-L76F, pCB-PL-W87R in the F-box-like motif, pCB-PL-G139RRR in the G139/W140/G141-like motif, and pCB-PL-F220R in the C-terminal conserved region) were successfully generated by reverse PCR with specific primers (Table 1).

### 3.3. Impact of VSR Defective Mutants on PLRV Accumulation in Inoculated N. benthamiana Leaves

**Figure 4:**
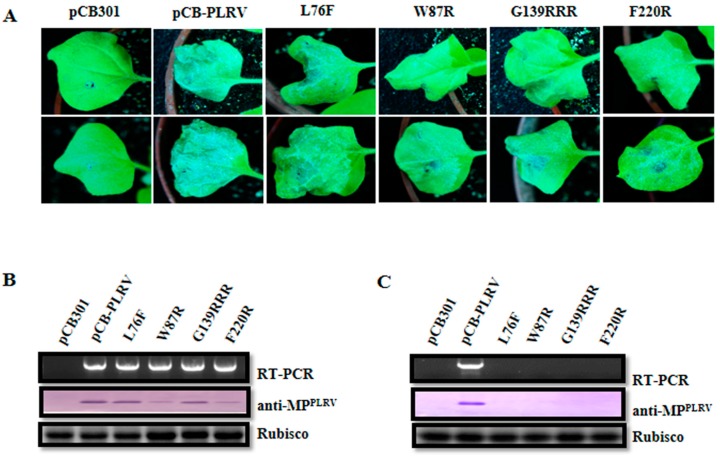
Infectivity analysis of the VSR defective mutants in F-box-like motif, G139/W140/G141-like motif, and the C-terminal conserved region derived from the full-length infectious cDNA clone of PLRV (pCB-PLRV) in *N. benthamiana*. (**A**) Symptoms on *N. benthamiana* leaves agroinfiltrated with pCB empty vector (negative control), wild-type pCB-PLRV (positive control) and mutants pCB-PL-L76F, pCB-PL-W87R, pCB-PL-G139RRR, and pCB-PL-F220R respectively, at 7 dpi. (**B**) Assessment of the viral RNA and protein accumulation in *N. benthamiana* leaves inoculated with pCB-PLRV mutants at 3 dpi by RT-PCR amplification and western blot analysis. (**C**) Assessment of viral RNA and protein accumulation in systemic leaves of *N. benthamiana* plants inoculated with pCB-PLRV mutants at 14 dpi by RT-PCR amplification and western blot analysis. The upper panel shows the RT-PCR amplification with PLRV-specific primer pair. The middle panel shows the western blot analysis with the PLRV-MP specific antibody (anti-MP^PLRV^). Rubisco stained with Coomassie brilliant blue used as a loading control is shown in the lower panel.

To investigate the impact of the above VSR defective mutants on the virus accumulation in inoculated leaves, *N. benthamiana* plants were agroinfiltrated at the four-leaf stage. The agroinfiltrated leaves with pCB-PLRV mutants, as well as wild-type pCB-PLRV (positive control), showed cell death symptoms at 7 dpi, whereas pCB empty vector (negative control) showed no cell death (Figure 4A). Inoculated leaves were collected at 3 dpi, and extracts subjected to RT-PCR and western blotting. The RT-PCR revealed that all pCB-PLRV mutants and wild-type pCB-PLRV could accumulate viral RNA in inoculated leaves, except for pCB empty vector (Figure 4B). Western blotting confirmed that wild-type pCB-PLRV and the mutants pCB-PL-L76F and pCB-PL-G139RRR accumulated viral protein at a relatively high level in inoculated leaves compared to mutants pCB-PL-W87R and pCB-PL-F220R.

To further investigate the effects of additional VSR on accumulation level of these VSR defective mutants, wild-type pCB-PLRV and the VSR defective mutants were respectively co-infiltrated into *N. benthamiana* leaves with P0^PL^. The leaves co-infiltrated with P0^PL^ and pCB-PLRV mutants showed more prominent cell death symptoms than infiltrated leaves with only pCB-PLRV mutants (without P0^PL^) at 7 dpi (Figure S2A). At 3 dpi of co-infiltration, RT-PCR and western blotting revealed that all mutants accumulated both RNA and proteins at similar levels to the wild-type (Figure S2B).

### 3.4. Impact of VSR Defective Mutants on PLRV Systemic Infection in N. benthamiana

Systemic leaves from inoculated *N. benthamiana* plants were harvested at 14 dpi and extracts were subjected to RT-PCR and western blotting. The RT-PCR and western blotting revealed that only the wild-type pCB-PLRV could systemically infect leaves, whereas all mutants from the three critical regions and pCB empty vector were unable to infect upper leaves (Figure 4C), indicating that all three motifs in the P0 (F-box-like motif, G139/W140/G141-like motif, and the C-terminal conserved region) were required for systemic infection of PLRV in *N. benthamiana*. Co-infiltration of all VSR defective mutants with a VSR (P0^PL^) also did not result in successful infection in upper leaves (Figure S2C).

### 3.5. Impact of VSR Defective Mutants on Virus Infection in Natural Hosts

**Figure 5:**
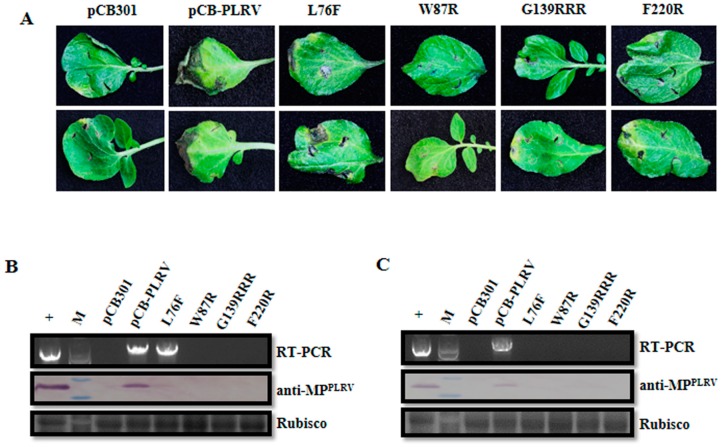
Infectivity analysis of VSR defective mutants in F-box-like motif, G139/W140/G141-like motif, and C-terminal conserved region derived from the pCB-PLRV in potato plants. (**A**) Symptoms on potato (cultivar Lalpakri) leaves agroinfiltrated with pCB empty vector (negative control), wild-type pCB-PLRV (positive control), and mutants pCB-PL-L76F, pCB-PL-W87R, pCB-PL-G139RRR, and pCB-PL-F220R, respectively, at 5 dpi. (**B**) Assessment of the viral RNA and protein accumulation in potato leaves inoculated with pCB-PLRV mutants by RT-PCR amplification and western blot analysis at 3 dpi. (**C**) Assessment of viral RNA and protein accumulation in systemic leaves of potato plants inoculated with pCB-PLRV mutants by RT-PCR amplification and western blot analysis at 14 dpi. Extracts from the inoculated *N. benthamiana* leaves were used as a positive control. The upper panel shows the RT-PCR amplification with PLRV-specific primer pair. The middle panel shows the western blot analysis with the PLRV-MP specific antibody (anti-MP^PLRV^). Rubisco stained with Coomassie brilliant blue used as a loading control is shown in the lower panel.

**Figure 6:**
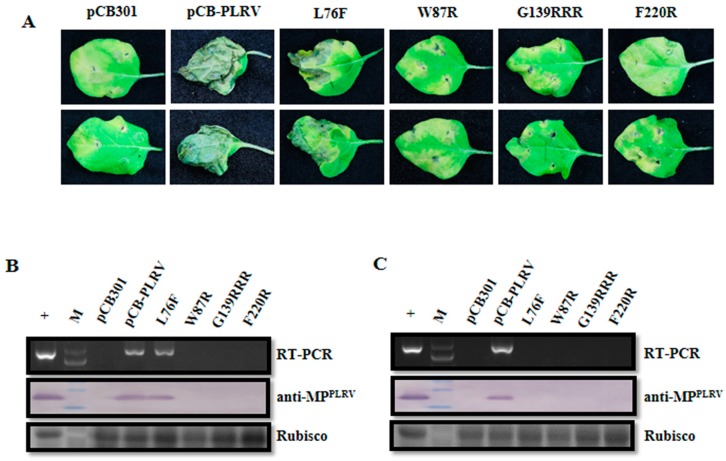
Infectivity analysis of the VSR defective mutants in F-box-like motif, G139/W140/G141-like motif, and C-terminal conserved region derived from the pCB-PLRV in black nightshade plants. (**A**) Symptoms on black nightshade leaves agroinfiltrated with pCB empty vector (negative control), wild-type pCB-PLRV (positive control) and mutants pCB-PL-L76F, pCB-PL-W87R, pCB-PL-G139RRR and pCB-PL-F220R respectively, at 5 dpi. (**B**) Assessment of viral RNA and protein accumulation in potato leaves inoculated with pCB-PLRV mutants by RT-PCR amplification and western blot analysis at 3 dpi. (**C**) Assessment of viral RNA and protein accumulation in systemic leaves of potato plants inoculated with pCB-PLRV mutants by RT-PCR amplification and western blot analysis at 14 dpi. Extracts from inoculated *N. benthamiana* leaves were used as a positive control. The upper panel shows the RT-PCR amplification with PLRV-specific primer pair. The middle panel shows the western blot analysis with the PLRV-MP specific antibody (anti-MP^PLRV^). Rubisco stained with Coomassie brilliant blue used as a loading control is shown in the lower panel.

Four- to five-week-old potato and black nightshade plants were respectively agroinfiltrated with the above-mentioned pCB-PLRV mutants to address the effect of these VSR defective mutants on PLRV infection in natural hosts. Potato and black nightshade leaves agroinfiltrated with pCB-PL-L76F mutant as well as wild-type pCB-PLRV showed cell death symptoms at 5 dpi, whereas the other three mutants and pCB empty vector showed no such symptoms (Figure 5A and Figure 6A). The RT-PCR and western blot for inoculated leaves at 3 dpi revealed that only the pCB-PL-L76F mutant and the wild-type pCB-PLRV accumulated in inoculated leaves, whereas all the other mutants did not (Figure 5B and Figure 6B). Western blotting showed that the virus accumulation level for the pCB-PL-L76F mutant was very low compared with wild-type pCB-PLRV. However, RT-PCR and western blotting showed that only the wild-type successfully systemically infected leaves of potato and black nightshade at 14 dpi (Figure 5C and Figure 6C), similar to the results for *N. benthamiana*.

## 4. Discussion

The P0 protein of poleroviruses suppresses the plant’s RNA silencing activity. The Inner Mongolian PLRV P0 protein (P0^PL^) is a potent suppressor of RNA silencing [30] and the suppressor activity is affected by substitutions in the F-box-like motif, G139/W140/G141-like motif, and C-terminal conserved region. However, F-box and G139/W140/G141 mutants had disturbed P1 amino acid sequence at the virus genome structure level. In this study, we further confirmed that L76F and W87R substitutions in the F-box-like motif and G139RRR substitution in the G139/W140/G141-like motif of P0^PL^ that did not affect the P1 amino acid sequence could abolish both local and systemic RNA silencing suppressor activity (Table S1). Similar observations were made by Pazhouhandeh et al. for P0^BW^LP1 and P0^CA^LP1 substitutions [33]. By the co-expression with additional suppressors of RNA silencing, TBSV P19 or P0^PL^, we observed significantly increased protein accumulation in respective co-infiltrated *N. benthamiana* leaves, indicating that P0 mutant proteins were stable in the presence of VSR [30]. Therefore, the available amino acid substitutions in the P0 essential motifs required for VSR activity without disturbing the P1 amino acid sequence were ready for further mutant construction in the full-length infectious clone of PLRV.

Subsequently, we generated VSR defective mutants in all three critical regions (pCB-PL-L76F and pCB-PL-W87R in the F-box-like motif, pCB-PL-G139RRR in the G139/W140/G141-like motif, and pCB-PL-F220R in the C-terminal conserved regions) of full-length infectious cDNA of PLRV (pCB-PLRV) to determine the effect of these mutants on PLRV infectivity. Detection of agroinfiltrated *N. benthamiana* leaves at 3 dpi demonstrated that all mutants could infect the inoculated leaves. However, the virus accumulation level of the mutants was lower than that of wild-type and they could not systemically infect leaves. Low levels of virus accumulation in inoculated leaves and the loss of systemic infection for these mutants may be due to the loss of silencing suppressor activity of P0 protein of PLRV. This result is compatible with the results of Pazhouhandeh et al. in which the CABYV LP2 mutant in the F-box accumulated ~10-fold less than the wild-type virus in agroinoculated *Arabidopsis* [46]; mutations in the 5′ terminal of BWYV ORF0 reduced virus accumulation 5–7-fold than the wild-type in agroinoculated *Nicotiana clevelandii* [38]. Interestingly, when these VSR defective mutants were co-infiltrated into *N. benthamiana* leaves with additional VSR (P0^PL^), they rescued the accumulation in inoculated leaves but could not produce systemic infection. These investigations revealed that the VSR protein of PLRV (P0^PL^) may act with other viral proteins or host proteins for systemic infection; however, the mechanism remains unknown and will be investigated in our future studies.

More interestingly, our results demonstrated that only the pCB-PL-L76F mutant could accumulate at a very low concentration in agroinoculated potato leaves, the principal host of PLRV rather than *N. benthamiana* at 3 dpi, whereas all other mutants could not accumulate. None of the mutants could infect upper leaves. Derrick and Barker also observed the reduced accumulation of PLRV in potato cultivars than that of *N. benthamiana* [47]. Sadowy et al. agroinoculated potato leaf disks with pBOK containing a full-length cDNA of PLRV and two P0 mutants: pBOK-T that contains an additional C-residue at nt 81 and pBQ47stop that changes Gln-47 of the putative ORF0 product (208–210 nt) into a stop codon [48]. Their RNA and protein analysis of the agroinoculated leaf disks showed that only pBOK could accumulate and the two P0 mutants failed to accumulate in the leaf disks, indicating that P0 was required for virus accumulation. However, earlier studies were on P0 null mutants, whereas we used VSR defective substitution mutants of PLRV. In our study, we also observed that only the pCB-PL-L76F mutant and wild-type pCB-PLRV could accumulate in the agroinoculated black nightshade leaves at 3 dpi. Interestingly, western blot revealed that the virus accumulation levels in agroinoculated black nightshade leaves were a little lower than that of agroinoculated *N. benthamiana* leaves, consistent with the results of Alvarez and Srinivasan [49], but higher than that of agroinfiltrated potato leaves. Similarly, in the case of systemic leaves, only the wild-type pCB-PLRV could accumulate at 14 dpi. Similar to VSR defective mutants of PLRV in this study, *Cucumber mosaic virus* (CMV) 2b (silencing suppressor protein) mutants were unable to systemically infect cucumber plants by preventing the systemic movement of RNA silencing signals [50,51]. Therefore, VSR is important for virus accumulation and systemic infection. Our results further demonstrated that the VSR defective mutants affected virus accumulation in inoculated leaves, which was clearly a host-dependent phenomenon because all the defective mutants could accumulate in *N. benthamiana*, whereas in potato and black nightshade, only the pCB-PL-L76F mutant accumulated at a significantly lower concentration.

## 5. Conclusions

Our study revealed that the VSR defective mutants of PLRV in the F-box-like motif, the G139/W140/G141-like motif, and the C-terminal conserved region of P0 protein affected virus accumulation in inoculated leaves and systemic movement in both *N. benthamiana* plants and its natural host, suggesting that the loss of systemic infection of these mutants may be due to the loss of VSR activity of P0 protein. However, there are many questions to be answered. Why did the VSR defective mutants have different accumulation levels in the inoculated leaves of *N. benthamiana* and its natural hosts, potato and black nightshade? Why were these VSR defective mutants with additional heterologous expression of P0^PL^ unable to successfully infect the upper leaves? The reasons for these phenomena will be addressed in our future investigations.

## Figures and Tables

**Table 1:** Primer sequences used in this study.

Primer	Sequence (5′ to 3′)
PLP0L76F-F	TTTCCGAGGCACCTCCACTA
PLP0L76F-R	TTGAAGGCCGGATGTTGAAA
PLP0W87R-F	CGGGGATTACTCTGCGGCAC
PLP0W87R-R	CTCAAGGCACTCATAGTGGA
PLP0F220R-F	CGAAGAACACTTACCGGTTT
PLP0F220R-R	AGACTTAGCGCGCCCTTGTA
P0G139F-F	TTTTGGGGACATGACATGGA
P0G139R-F	CGTTGGGGACATGACATGGA
P0W140R-F	GGTAGGGGACATGACATGGA
P0G141F-F	GGTTGGTTTCATGACATGGA
P0G141R-F	GGTTGGCGACATGACATGGA
P0G139F-R	GTTTGACAATCCAGCCGCAT
P0G139C-F	TGTTGGGGACATGACATGGA
P0G139S-F	AGTTGGGGACATGACATGGA
P0G140G-F	CGTGGGGGACATGACATGGA
PLP0G139CR-F	TGTAGGGGACATGACATGGA
PLP0G139CRR-F	TGTAGGCGACATGACATGGA
PLP0G139FR-F	TTTAGGGGACATGACATGGA
PLP0G139RR-F	CGTAGGGGACATGACATGGA
PLP0G139RRR-F	CGTAGGCGACATGACATGGA
PLRV5-28F	ACAAAAGAATACCAGGAGAAATTGCAGC
PLRVKp3R	AAGGTACCACTACACAACCCTGTAA
PLRV2723F	CTTCAAAAGGTGTCAGGAG
PLRV3656R	GCCTGCGAAGGGATTG
pCB/Not I-F	GCGGCCGCGGTGTCTCGCAC
PLRV/Apa I-R	GGGCCCACGATTTGTATAGC
PLRV/Apa I-F	GGGCCCTACCATCGTCATTA
PLRV/Spe I-R	ACTAGTATGGAGATATCATT
M13F-47	CCAGGGTTTTCCCAGTCACGAC
M13R-48	AGCGGATAACAATTTCACACAG
BinF	GTAAGGGATGACGCACAATC
Pocon-F	GAYTGYTCYGGTTTTGACTGG
Pocon-R	TTRTAYTCATGGTAGGCCTTGAG

## Supplementary Materials

The following are available online at http://www.mdpi.com/1999-4915/11/2/170/s1. Figure S1. Infectivity analysis of full-length cDNA of pCa-PLRV and pCB-PLRV in *N. benthamiana*. (**A**) Symptoms on *N. benthamiana* leaves agr-infiltrated with pCB empty vector (negative control), pCa-PLRV, and pCB-PLRV, respectively, at 7 dpi. (**B**) Assessment of viral RNA accumulation in systemic leaves of inoculated *N. benthamiana* plants at 14 dpi by RT-PCR amplification with PLRV-specific primer pair. Figure S2. Impact analysis of additional heterologous expression of VSR (P0^PL^) on the infectivity of VSR defective mutants in F-box-like motif, G139/W140/G141-like motif, and C-terminal conserved region derived from pCB-PLRV in *N. benthamiana*. (**A**) Symptoms on *N. benthamiana* leaves agroinfiltrated with pCB empty vector (negative control), wild-type pCB-PLRV (positive control), and mutants pCB-PL-L76F, pCB-PL-W87R, pCB-PL-G139RRR, and pCB-PL-F220R, respectively, in absence or presence of P0^PL^ at 7 dpi. (**B**) Assessment of viral RNA and protein accumulation in *N. benthamiana* leaves inoculated with pCB-PLRV mutants in absence or presence of P0^PL^ at 3 dpi by RT-PCR amplification and western blot analysis. (**C**) Assessment of viral RNA and protein accumulation in systemic leaves of *N. benthamiana* plants inoculated with pCB-PLRV mutants in absence or presence of P0^PL^ at 14 dpi by RT-PCR amplification and western blot analysis. The upper panel shows the RT-PCR amplification with PLRV-specific primer pair. The middle panel shows the western blot analysis with the PLRV-MP specific antibody (anti-MP^PLRV^). Rubisco stained with Coomassie brilliant blue used as a loading control is shown in the lower panel. Table S1. Test results for RNA silencing suppression of P0^PL^ and its mutants.
