# Supplementary material for: The Three Essential Motifs in P0 for Suppression of RNA Silencing Activity of Potato leafroll virus Are Required for Virus Systemic Infection
Source: Viruses. 2019 Feb 20;11(2):170. doi: 10.3390/v11020170 (PMC6410027; doi:10.3390/v11020170)
Supplement: Supplementary file 1 [file viruses-11-00170-s001.zip › Supplementary materials/Table S1.docx]

**Table S1.** Test results for RNA silencing suppression of P0^PL^ and its mutants.

| **Important motifs** | **P0^PL^ mutants** | **The position of substitutions in the amino acid sequence** | **Local RNA silencing suppression** | **Systemic RNA silencing suppression** |  |
| --- | --- | --- | --- | --- | --- |
| **F box like motif** | **Wild-type** | 76-LPRHLHYECLEWGLLCGTHP-95 | + | + | This study |
|  | L76F | **F**PRHLHYECLEWGLLCGTHP | - | - |  |
|  | W87R | LPRHLHYECLE**R**GLLCGTHP | - | - |  |
| **G139/W140/G141 like motif** | **Wild-type** | 131-QNAAGLSNGWGHDMEAF-147 | + | + |  |
|  | G139F | QNAAGLSN**F**WGHDMEAF | + | + |  |
|  | G139R | QNAAGLSN**R**WGHDMEAF | + | nd |  |
|  | W140R | QNAAGLSNG**R**GHDMEAF | + | nd |  |
|  | G141F | QNAAGLSNGW**F**HDMEAF | + | nd |  |
|  | G141R | QNAAGLSNGW**R**HDMEAF | + | nd |  |
|  | G139C | QNAAGLSN**C**WGHDMEAF | + | nd |  |
|  | G139S | QNAAGLSN**S**WGHDMEAF | + | nd |  |
|  | W140G | QNAAGLSNG**G**GHDMEAF | + | nd |  |
|  | G139CR | QNAAGLSN**CR**GHDMEAF | + | nd |  |
|  | G139FR | QNAAGLSN**FR**GHDMEAF | + | + |  |
|  | G139RR | QNAAGLSN**RR**GHDMEAF | + | nd |  |
|  | G139CRR | QNAAGLSN**CRR**HDMEAF | + | + |  |
|  | G139RRR | QNAAGLSN**RRR**HDMEAF | - | - |  |
| **C-terminal conserved region** | **Wild-type** | 220-FRTLTGFPIYVPSEDYLEGS-239 | + | + | Zhou et al., 2014 |
|  | F220R | **R**RTLTGFPIYVPSEDYLEGS | - | - |  |

+: Retain; -: Abolish; nd: not examine
