# Supplementary figures and images for: The Three Essential Motifs in P0 for Suppression of RNA Silencing Activity of Potato leafroll virus Are Required for Virus Systemic Infection
Source: Viruses. 2019 Feb 20;11(2):170. doi: 10.3390/v11020170 (PMC6410027; doi:10.3390/v11020170)

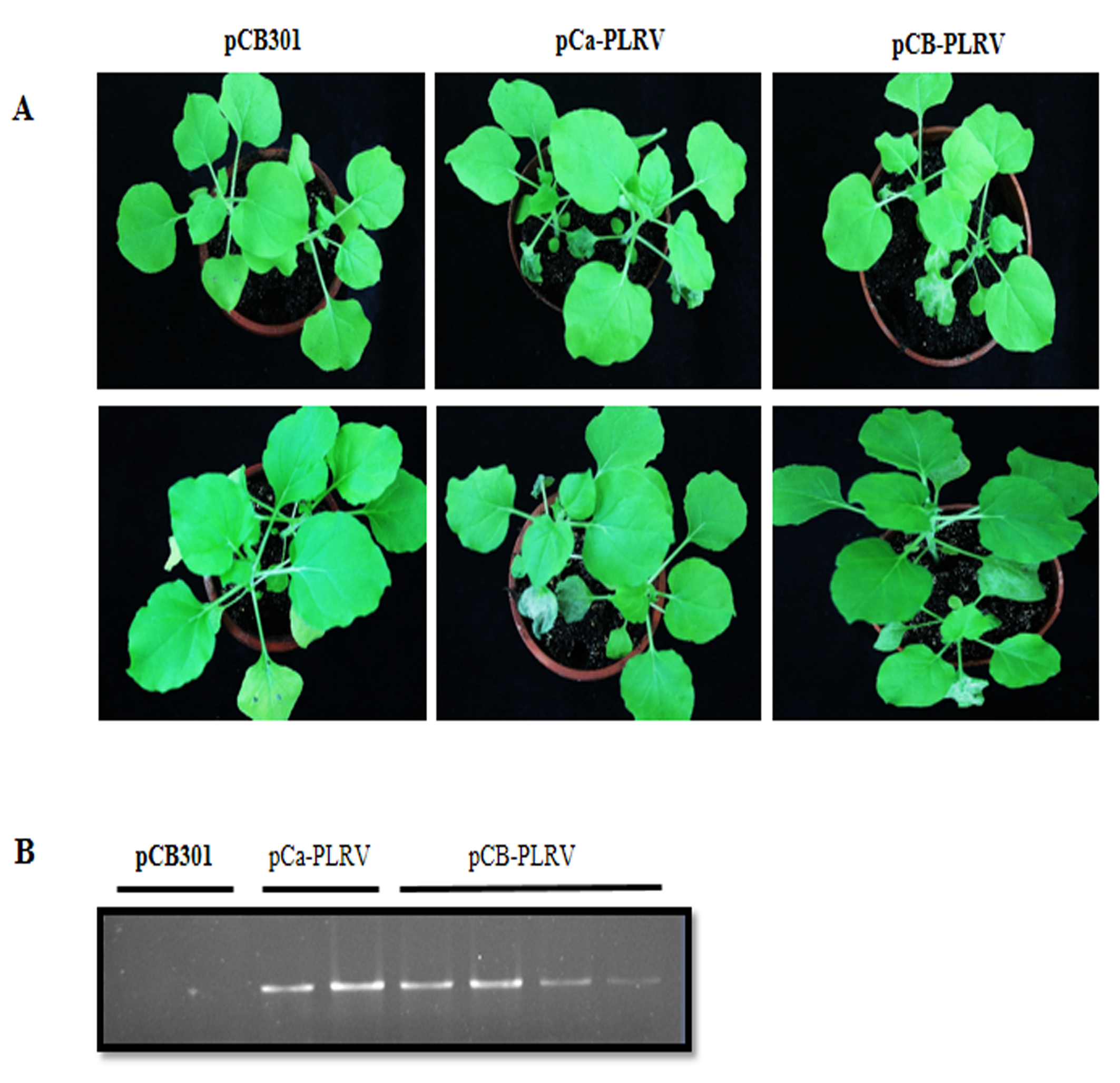

Supplement: Supplementary file 1 [file viruses-11-00170-s001.zip › Supplementary materials/Figure S1.tif]

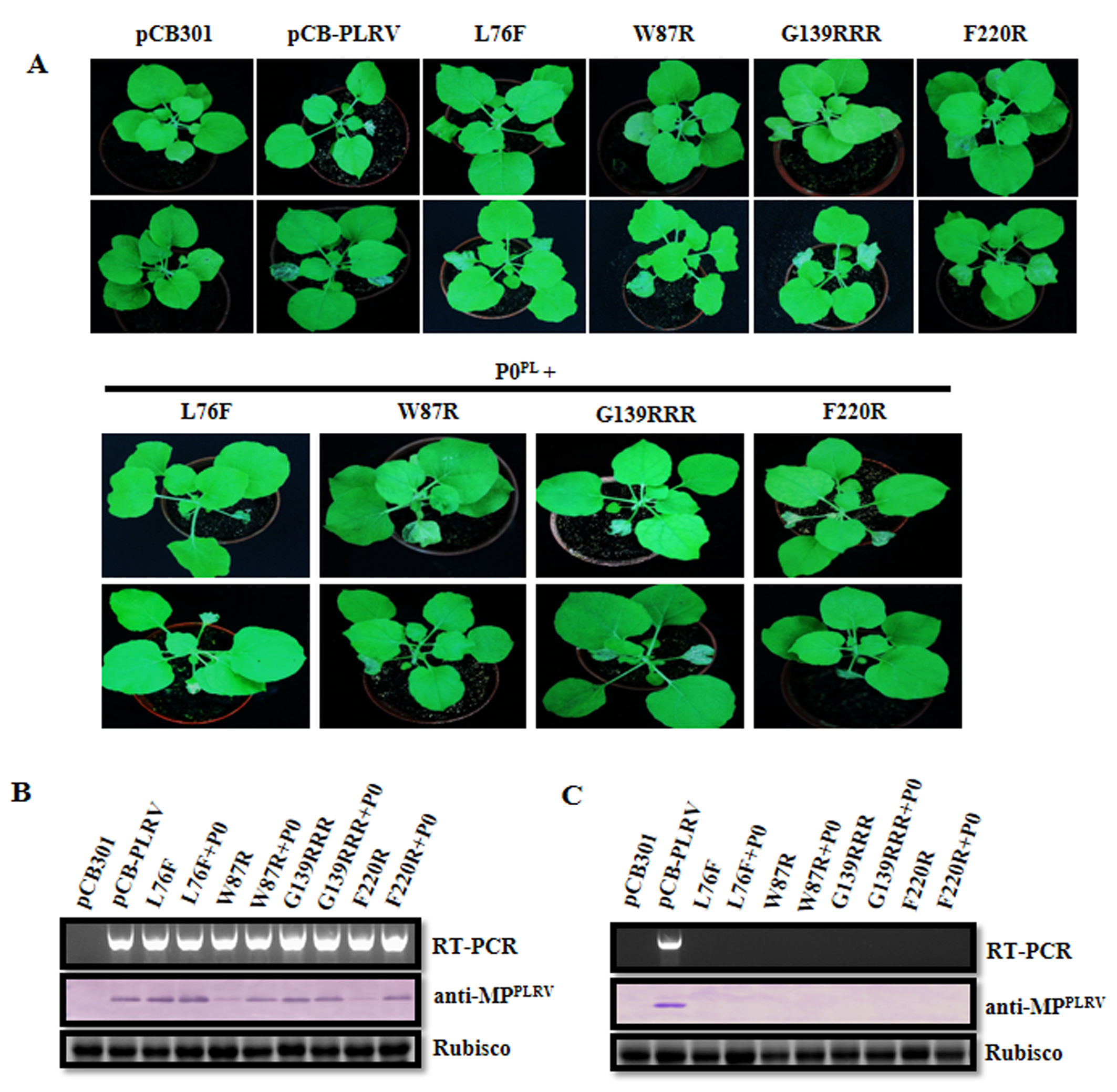

Supplement: Supplementary file 1 [file viruses-11-00170-s001.zip › Supplementary materials/Figure S2.tif]
